# Parameter inference for discretely observed stochastic kinetic models using stochastic gradient descent

**DOI:** 10.1186/1752-0509-4-99

**Published:** 2010-07-21

**Authors:** Yuanfeng Wang, Scott Christley, Eric Mjolsness, Xiaohui Xie

**Affiliations:** 1 Department of Physics and Astronomy, University of California, Irvine, CA 92617, USA; 2 Department of Mathematics, University of California, Irvine, CA 92617, USA; 3 Department of Computer Science, University of California, Irvine, CA 92617, USA; 4 Center for Complex Biological Systems, University of California, Irvine, CA 92617, USA; 5 Institute for Genomics and Bioinformatics, University of California, Irvine, CA 92617, USA

## Abstract

**Background:**

Stochastic effects can be important for the behavior of processes involving small population numbers, so the study of stochastic models has become an important topic in the burgeoning field of computational systems biology. However analysis techniques for stochastic models have tended to lag behind their deterministic cousins due to the heavier computational demands of the statistical approaches for fitting the models to experimental data. There is a continuing need for more effective and efficient algorithms. In this article we focus on the parameter inference problem for stochastic kinetic models of biochemical reactions given discrete time-course observations of either some or all of the molecular species.

**Results:**

We propose an algorithm for inference of kinetic rate parameters based upon maximum likelihood using stochastic gradient descent (SGD). We derive a general formula for the gradient of the likelihood function given discrete time-course observations. The formula applies to any explicit functional form of the kinetic rate laws such as mass-action, Michaelis-Menten, etc. Our algorithm estimates the gradient of the likelihood function by reversible jump Markov chain Monte Carlo sampling (RJMCMC), and then gradient descent method is employed to obtain the maximum likelihood estimation of parameter values. Furthermore, we utilize flux balance analysis and show how to automatically construct reversible jump samplers for arbitrary biochemical reaction models. We provide RJMCMC sampling algorithms for both fully observed and partially observed time-course observation data. Our methods are illustrated with two examples: a birth-death model and an auto-regulatory gene network. We find good agreement of the inferred parameters with the actual parameters in both models.

**Conclusions:**

The SGD method proposed in the paper presents a general framework of inferring parameters for stochastic kinetic models. The method is computationally efficient and is effective for both partially and fully observed systems. Automatic construction of reversible jump samplers and general formulation of the likelihood gradient function makes our method applicable to a wide range of stochastic models. Furthermore our derivations can be useful for other purposes such as using the gradient information for parametric sensitivity analysis or using the reversible jump samplers for full Bayesian inference. The software implementing the algorithms is publicly available at http://cbcl.ics.uci.edu/sgd

## Background

It is becoming increasingly apparent that stochasticity, whether intrinsic or extrinsic, plays an important role in the dynamics and behavior of biological systems. In systems biology and the study of gene expression [1-3], the consequences of stochasticity can manifest in numerous ways such as slow promoter kinetics leading to gene transcription bursting [4,5], finite-number effects and mRNA translation bursting [6-9], propagation of noise in gene regulatory cascades [4,10], and phenotypic switching [11,12]. In some cases, biological systems evolve to minimize the effects of noise such as through negative feedback loops [13-15], but there is also evidence that biology exploits randomness such as to create phenotypic diversity in populations thus allowing better adaptation to changing environments [16-18]. With the growing awareness of stochasticity in biology and the increasing use of stochastic models in computational systems biology, there is a need to develop new analysis and computational techniques for studying, understanding and designing these stochastic models.

One particular analysis technique and challenge in computational systems biology is the inference of rate parameters from experimental data for a specified biochemical system [19]. Parameter inference for continuous deterministic models has a considerable body of research literature and can often be converted into an optimization problem for which many computational methods are available [20]. The strategies of these methods can be classified as either deterministic or stochastic. Deterministic strategies are generally only applicable for specific mathematical formulations of the model where a statement about the existence of the global optimum can be guaranteed along with a constructive algorithm to find it. Many problems are not that well-defined so stochastic strategies are popular including stochastic gradient descent [21], simulated annealing [22-24], evolutionary computation [25], and other heuristics. Regardless, considerable computational effort is required for all of these methods as many simulations of the continuous deterministic model are performed. A discrete stochastic model is essentially a more adequate description for a biochemical system, but it has the disadvantage of being computationally expensive to simulate as well as requiring numerous independent simulations to be performed in order to calculate expectation values of various model outputs [26-28]. These computational challenges mean that approximation techniques are frequently used for parameter inference including simplification of the stochastic model [29] and approximate inference such as using the chemical Langevin equation [30] in place of the Markov jump process [31,32]. Recent research has shown that parameter inference for stochastic models is feasible given time course observations of the system, even if only a partial set of molecular species are observed [32,33]. However the current algorithms, based on the Bayesian framework, are typically time-consuming due to the need of sampling high dimensional space. Therefore there are significant challenges in applying the method to real systems, such as gene regulatory networks [34].

Most proposed methods for parameter inference in stochastic biochemical models consider how to calculate the maximum likelihood for the rate parameter values given a stochastic model and observational data. Except for the simple models, the likelihood function is computationally intractable, so these methods either perform exact inference on an approximated model where the likelihood computation is tractable, or they approximate the likelihood with a more tractable function, or some combination of the two. Tian *et al. *[35] considered the simulated maximum likelihood (SML) method that estimates likelihood by generating samples from many simulations of the stochastic model. The ratio of samples matching observations to the total number of samples is used to estimate the transitional density and the log-likelihood. Then a genetic algorithm is used to obtain the optimal rate parameter values that minimize the log-likelihood function. While the SML approach is straightforward, it is computationally expensive because it requires a large number of simulations of the stochastic model. Similarly, approximate Bayesian computation also requires the stochastic model to be simulated, but it avoids calculating the likelihood function by comparing simulated data with observations using a rejection sampler [36,37]. In a similar framework, Yosiphon et al. [38] used a simulated annealing procedure in an MCMC algorithm to estimate the parameters in stochastic models of reaction networks. Reinker et al. [39] proposed a method utilizing a hidden Markov model to approximate the stochastic model that takes observational error into account. Boys et al. [33] showed how full Bayesian inference can be performed on the stochastic Lotka-Volterra model along with performance of various Markov chain Monte Carlo (MCMC) algorithms. Interestingly they showed that with partially observed data, i.e., only one of the two species in the model, they can still make inferences about all three rate parameters in the model; though it is unclear how well this would work on larger models with many parameters. Wilkinson and colleagues have investigated additional methods including using diffusion approximations [29,31] and incorporating multiple data sources [40].

In this paper, we describe an alternative method for parameter inference in discretely observed stochastic kinetic models. Instead of calculating and approximating the likelihood function as in the previous methods, we focus on estimating the gradients of the likelihood function with respect to the parameters. In particular, we propose a general methodology for efficiently estimating the gradients using reversible jump Markov chain Monte Carlo (RJMCMC). RJMCMC is an extension of the standard MCMC method that allows for generating samples on spaces of varying dimensions [41]. An implementation challenge for RJMCMC is the lack of a general way to construct the jump proposals such that detailed balance is preserved [42]. For stochastic kinetic models, the jump proposal corresponds to moves that change the number and the time of reactions that occur between two observations of the system. For most models, there is an infinite set of possible reaction processes (constrained by the observation data) that can occur between two time points, and the probabilities of different reaction paths depend upon the rate parameter values. Utilizing the research in flux balance analysis for metabolic networks [43-45], we provide an algorithm so that jump proposals can be automatically constructed from any standard biochemical model, thus allowing RJMCMC to be used without requiring any manual analysis by the modeler.

The availability of the gradient information allows for inference of the rate parameters of stochastic kinetic models using gradient descent-based methods. We implement a steepest gradient descent method for parameter inference using the estimated gradient information in a MATLAB software package http://cbcl.ics.uci.edu/sgd. We demonstrate the utility of our algorithms using two example stochastic models, including a birth-death process and a gene auto-regulation model.

## Methods

### Stochastic kinetic model of reaction systems with discrete states

Consider a general reaction system involving *M *reactions *R*_*1*_, *R*_*2*_,..., *R*_*M *_and *K *species *S*_*1*_, *S*_*2*_,..., *S*_*K*_. We denote the state of the reaction system by *X *= (*x*_1_,⋯,*x*_*K*_) where *x*_*a *_is the number of species *S*_*a*_. Each reaction *R*_i _has an associated rate law, represented by a hazard function *h*_*i*_(*X*, Θ) (also called rate function), where Θ ≡ {*θ*_*r*_} is a set of parameters associated with the reactions. Suppose the reaction system takes the following form(1.1)

where *u*_*ra *_and *v*_*ra *_are the positive integer stoichiometries associated with reaction *R*_*r *_for reactant *S*_*a*_, representing the amount of species *S*_*a *_that decrease and increase respectively when reaction *R*_*r *_occurs. Eq. (1.1) can be represented more compactly as *US *→ *VS*, where *U *= [*u*_*ij*_] and *V *= [*v*_*ij*_] are *M *× *K *matrices. We define the net effect reaction matrix *A *= *V - U*, which reflects the net change of species numbers associated with reactions.

Asumming the reaction system is in well-stirred condition with a fixed volume, we can introduce the master equation model, also known as "chemical master equation (CME)" in the biochemical modeling field [26], which describe the time evolution of the state probability using a set of ordinary differential equations. The CME can be derived for any biochemical reaction system using the standard continuous time Markov process theory. Denote *P*(*X*;*t*) the probability of the system in state *X *at time *t*. For an infinitesimal time increment Δ*t*, *P*(*X*;*t *+ Δ*t*) can be written as the sum of probabilities of the number of ways in which the system can reach or leave the current state:(1.2)

where *A*_*i *_denotes the *i*th row of the net effect matrix *A*, and *h*_*i*_(*X*, Θ) is the hazard function, determining the rate of probability transition out of state *X *due to reaction type *i*. In the limit of Δ*t *→ 0, Eq. (1.2) adopts the standard master equation form,(1.3)

with(1.4)

Suppose all possible system states (usually countably infinite) are ordered and represented by indices 1, 2,..., etc. Then Eq. (1.3) can be rewritten as , where  is a row vector with *P*_*i*_(*t*) representing the probability of the *i*-th state at time *t*.

For reactions that obey mass-action law kinetics, one rate parameter *θ*_*r *_is associated with each reaction type *i*, and consequently the hazard function has the form of(1.5)

where *u*_*ra *_is the stoichiometry coefficient of reactants *a *in reaction *R*_*r*_. Forms of other rate laws for chemical kinetics, e.g. the Michaelis-Menten model, can be found in [46]. Although we will focus our discussion on the hazard function in the form of Eq.(1.5), the following analysis can handle more general cases as long as the explicit functional form of the hazard function is known.

### Gradient of the likelihood function with discrete observations

Our goal is to estimate the rate parameters of a stochastic model based on the observations at a set of discrete time points. Suppose we have observations {*X*_Γ_(*t*_1_), *X*_Γ_(*t*_2_),⋯,*X*_Γ_(*t*_*m*_)} of the system at *m *discrete time points {*t*_1_, *t*_2_,...,*t*_*m*_} for a subset of species Γ ⊆ {1,⋯,*K*}. We say the system is fully observed if Γ = {1,⋯,*K*}, and partially observed otherwise. Denoting the likelihood of the observations for a given set of rate parameters by *L*(*X*_Γ_(*t*_1_), *X*_Γ_(*t*_2_),⋯,*X*_Γ_(*t*_*m*_); Θ), we estimate the rate parameters by maximizing the likelihood function.

For simplicity of discussion, consider first a single time interval [*t*_s_, *t*_*s*+1_] with full observations available at the start and the end of the interval, denoted by *X*(*t*_s_) and *X*(*t*_*s*+1_) respectively. Let *L*(*X*(*t*_*s*_), *X*(*t*_*s*+1_);Θ) denote the likelihood of observing *X*(*t*_s_) and *X*(*t*_*s*+1_) under a model with parameters Θ. In Appendix, we show that the gradient of the likelihood function with respective to parameters can be calculated using the following formula, for any stochastic system with a master equation Eq. (1.3)(2.1)

where *T*_k _is the time duration of the system at state *k*, and *N*_*k, k' *_is the number of transitions from state *k *to *k' *occurred during the interval. Both *T*_k _and *N*_*k, k' *_are random variables, and can be viewed as the sufficient statistics of the model. *E*[·] represents the expectation of the random variables. The formula suggests that we can calculate the gradient of the likelihood function by estimating the expectations of the two sufficient statistics.

For the biochemical reaction system in Eq. (1.1), suppose *J *reactions have occurred during the time interval [*t*_*s*_, *t*_*s*+1_] with the types and the corresponding times of the reactions denoted by . Then by Eq. (2.1), the gradient of the likelihood function can be rewritten as(2.2)

where , which is fully specified by Ξ, denotes the state of the system between , and . Eq. (2.2) can also be written in an alternative form(2.3)

where

is the likelihood of the reaction process Ξ. If all the reactions follow mass-action law in Eq.(1.5), the gradient formula can be further written as(2.4)

Now return to the general case where the observations are available at multiple time points from a subset of the species. The above formula for calculating gradient can still hold if we view the entire duration of the observations as a single time interval. However, the expectation in Eq. (2.3) is now taken on the systems states whose distribution is conditioned on the observations at the intermediate time points.

In general, the expectation in Eq. (2.3) cannot be calculated exactly. Instead we utilize a sampling method to approximate the expectation. More specifically, we sample the latent path conditioned on the parameters and the observations, and then calculate the quantity in Eq. (2.3) by averaging over the sampled paths to obtain the gradient. The same strategy also applies to the partially observed case, as long as the reaction paths are sampled conditioned on the partial observation data.

### Reversible jump Markov chain Monte Carlo sampling

To calculate the gradient, we need to find an efficient way to sample the latent reaction processes conditioned on the observations. One commonly used sampling method is the stochastic simulation algorithm (SSA) [26], which can be used as a rejection method to discard samples that do that match the end state. The SSA method is computationally inefficient for generating samples between two measurements when the total number of possible states is high (as in the case of the biochemical reactions), because the chance of a sampled trajectory matching the end state is typically small and consequently most of the samples will likely be rejected.

Here we use the framework of RJMCMC [41] to sample the latent process. RJMCMC is a generalized MCMC method that can construct a sampler between models of different dimensions, which in our case corresponds to reaction paths with different number of reactions. To sample latent paths in biochemical reaction systems, the RJMCMC method [33] first generates an initial reaction path that is consistent with the observations. Then RJMCMCM constructs a Markov chain by a) proposing a new sample path by adding or deleting a specific set of reactions from the current path, and b) determining whether to accept the new sample or keep the previous one according to an acceptance probability.

Therefore, to construct a RJMCMC sampler, we will need to consider three issues: 1) how to generate the initial path; 2) how to propose a set of reactions for addition or deletion; and 3) how to determine the acceptance probability of a new path. Note that both the initial path and the proposed path have to match the observations at the start and the end of the interval, implying that only a subset of the reactions can be used for either initialization or addition/deletion. While the RJMCMC sampler exists for some specific reaction systems [33], usually taking advantage of the domain-specific knowledge, the challenge, however, is to find a general method that can work for any arbitrary reaction system.

Next we address the three issues mentioned above, and describe a general method to automatically construct a RJMCMC sampler for an arbitrary reaction system.

#### 1) Generating an initial reaction path using integer programming

The first issue of generating the initial path is relatively easy to address. Let *r *be a vector representing the number of each reaction type occurred within the initial path. To match the observations at the start and the end of each interval, *r *has to satisfy certain constraints. Fortunately, all these constraints are linear, and thus we can use linear integer programming to find a solution. In practice, we used the GNU Linear Programming Kit (*GLPK *library) [47], which is incorporated into our MATLAB package using the interface *GLPKMEX *[48].

#### 2) Proposing a new sample by adding or removing reactions

After an initial path is generated, our next step is to use proposal moves to add or remove reactions. Before describing our method, we first introduce two concepts that are used in studying biochemical reaction systems.

#### Definition 1: Elementary Mode

*An elementary mode (EM) of a biochemical reaction network is a set of reactions that does not alter the observed number of molecular species. Formally, an elementary mode **is a column vector of non-negative integers that satisfies *, *where Ã = A ( the net-effect reaction matrix) when all species are observable, and is a sub-matrix of A with columns corresponding to the observed species when only a subset of species are observable*.

#### Definition 2: Null Set

*The null set is a set consisting of all independent elementary modes, denoted by *.

Note that the null set is usually different between the fully and the partially observed case because of the different *Ã *matrix used.

Elementary modes analysis is well studied in metabolic networks theory and is used to find the flux distribution of the metabolic network at a steady state [49]. Various tools have been developed to identify *EM*s [43-45]. In this work, we used the *metatool *package [44] to calculate the null set of any specific reaction network, which has been shown to be efficient for large networks.

Provided with a valid reaction path and the null set, we then proceed to generate a new sample by taking one of the following three move types. After randomly choosing an elementary mode  from the null set,

1. With probability α_1_, add the set of reactions in  with random reaction times uniformly distributed within the interval.

2. With probability α_2_, remove one set of randomly selected reactions in  from the current path within the interval.

3. With probability 1-α_1_-α_2_, randomly move the time of all reactions.

Using  ensures that the proposed reaction path is always consistent with the observations. However, there are two additional conditions for a new sample path to be valid: 1) the number of any reaction type must be positive after the move, and 2) the population numbers for all species remain positive throughout the whole process. If either of the two conditions is violated, we set the likelihood of the new sample path to be zero and reject the new sampled path. The proposal probability in RJMCMC for different moves is set be to α_1 _= α_2 _= 0.25 in practice. Note that the initial path and the null set only need to be calculated once, and thus they only impose a modest computational burden on the sampling algorithm.

#### 3) Determining acceptance probability

Next we address the third issue on how to determine the acceptance probability of a proposed sample. We discuss the fully observed case first, and then the partially observed case.

#### Fully observed case

The observations at *m *discrete time points break the entire observation window into *m*-1 subintervals. Because all species are observed, the reaction path at each sub-interval is completely independently of each other conditioned on the observations. The reaction path at each sub-interval can therefore be sampled independently using RJMCMC. Let Ξ denote the current reaction path and Ξ denote the proposed reaction path. The probability of accepting the new path is specified by min(1, *AR*_*p*_), with *p *= 1, 2, or 3 denoting the type of the move

where *π*(Ξ|Θ), defined in Eq. (2.3), is the likelihood of sample path Ξ, *r*_j _is the number of type *j *reaction in the current sample path, *q*_*k, j *_denotes the number of reaction type *j *in the elementary mode , and *τ *is the time length of the sub-interval. *Appendix, Algorithm 1 *provides the pseudo-code for the fully observed case.

#### Partially observed case

In the partially observed case, observations are only available for a subset of the species. Different from the fully observed case, the reaction paths at different sub-intervals are now correlated, caused by unobserved species. Consequently, RJMCMC can no longer be applied independently for each sub-interval.

To account for the correlation, we use a new strategy in which the reaction paths at two consecutive sub-intervals are sampled together at each sampling step using correlated moves. Let {*q*_*k*_'}, *k *∈ (1, *K'*) be the null set corresponding to the partially observed case. Note that adding/deleting the set of reactions in  only ensures that the observed species' numbers remain unchanged, but not the unobserved species. Suppose we are to update the reaction path following the time point *t*_*i*_. We first generate a new sample path in the i-*th *interval [*t*_*i*_, *t*_*i*+1_] using the same reversible jump moves as described for the fully observed case, with a randomly chosen elementary mode. If the move changes the unobserved species numbers at time *t*_*i*+1_, we subsequently update the (i+1)-*th *interval using a complementary move that keeps the system state at the end of the second interval unchanged. For example, if move type 1 (or 2) is chosen to update the first interval with an elementary mode *q'*_*k*_, move type 2 (or 1) will be applied to the second interval to remove (or add) the same elementary mode *q'*_*k*_. The complementary moves guarantee that the new reaction paths proposed for the two sub-intervals do not change the species numbers, including those of the unobserved species, at the end of the second interval. As with the fully observed case, the two conditions of a valid path (positive reaction type number and positive species number) must be satisfied, otherwise the proposal move will be rejected. The acceptance probability is calculated as , where *p*' denotes the complementary move type of *p*. In this way, the state of unobserved species at time *t*_*i *_(*i *= 2,⋯,*m*) can be updated sequentially. An additional step is used to update the state at the first observation time point *t*_1_, which is done by keeping the species number at the end of the first interval fixed and changing the start state according to the proposed move. *Appendix, Algorithm 2 *provides the pseudo-code of using RJMCMC for the partially observed case.

### Stochastic gradient descent algorithm

Given the estimated gradient of the likelihood function, we use the method of steepest descent to find an optimal solution of the parameters. At each step of the algorithm, we first generate sample paths using the RJMCMC algorithm at current parameter values. After burn-in, we calculate the gradient of the likelihood function using the formula in (2.4). The estimated gradient is then used to update the parameter values until convergence. A simple strategy for choosing the step size is to set it to be a constant. Although this works well for simple systems, it sometimes induces over-shooting of the parameter values or slow convergence during the gradient descent. When this happens, we adaptively adjust the step size within a certain range according to the gradient value. An overview of the stochastic gradient descent algorithm is given in *Appendix, Algorithm 3*.

## Results

Next we illustrate the utility of our algorithm using two example reaction systems. In both cases, we simulated the reactions of the system using the stochastic simulation algorithm, and recorded the species numbers at a set of discrete time points, which were treated as observations of the system. Our method was then applied to infer the rate parameters for each system based on these observations.

### Example 1: Birth-death process

We first applied our algorithm to a well-studied birth-death process, which can be seen as a simplified model of production and degradation of a single molecular species [34]. The reactions are

We assume that *R*_1 _and *R*_2 _follow the first-order and zeroth-order mass-action law respectively. Denote the number of *A *molecules by *n*_*A*_, thus the hazard function is given by *h*_1 _= *k*_1_*n*_*A *_and *h*_2 _= *k*_2_. The net-effect reaction matrix of the system is A = (-1, 1)^T^. Consequently, the null set of the system contains only one elementary mode , i.e. the combination of *R*_1 _and *R*_2_.

We generated observations by simulating the reaction process using SSA with different parameter sets (*k*_1_, *k*_2_) = (0.03,0.6), (0.06, 0.6), (0.1, 0.6), (0.1, 0.3) and (0.03, 0.2). For each parameter set, four observation datasets were generated that differ on the total observation time (*T*) and the observation interval (Δ*t*) (see Table 1).

**Table 1:** Parameter inference result for the birth-death model

*Dataset* (*m *Δ*t*)	(0.03 0.6)*	(0.06 0.6)*	(0.1 0.6)*	(0.03 0.1)*	(0.03 0.2)*
**(21 2)**	0.030 0.61	0.041 0.36	0.101 0.47	0.035 0.167	0.02 0.28
**(51 2)**	0.030 0.78	0.077 0.75	0.12 0.63	0.032 0.074	0.029 0.15
**(21 5)**	0.026 0.51	0.082 0.67	0.12 0.69	0.026 0.092	0.028 0.21
**(101 10)**	0.026 0.51	0.040 0.42	0.067 043	0.024 0.094	0.026 0.175

*True values of parameters

*m *: total number of observations; Δ*t*: the time between two observations.

**Figure 1:**
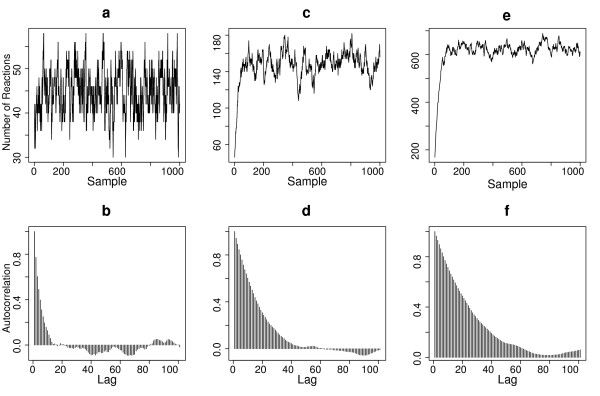
**Property of the RJMCMC sampler for the birth-death model**. Panel **a, c, **and **e **plot the total number of reactions in each of the samples generated by RJMCMC, corresponding to three different observation datasets with Δ*t *= 2, 5 and 10 respectively. Panel **b, d, **and **f **shows the autocorrelation on the number of reactions corresponding to panel **a, c, **and **e **respectively. The rate parameters used are (*k*_1_, *k*_2_) = (0.03, 0.6).

We first examined the convergence property of the RJMCMC sampler with the different datasets generated with the first parameter set. Figure 1 shows the trace plots and autocorrelations of the total number of reactions in the sample paths. In all cases, we found the RJMCMC sampler is efficient and induces good mixing of sample paths, with convergence occurring typically within 100 samples. As expected, larger correlation length is observed for data with longer observation intervals (Figure 1d and 1f).

We applied the SGD algorithm to estimate the two rate parameters for each dataset. The convergence criterion is set to be that the relative changes of all parameter values are less than 0.005. We used 1000 samples after a burn-in of 100 samples to estimate the gradient for a given set of parameter values. The estimated parameters for each dataset are summarized in Table 1. In all cases, the inferred parameters showed a good agreement with the true values, although the accuracy of the estimation clearly correlates with the number of observations and the observation time intervals. For datasets with larger observation interval and fewer data points, larger variation is the observed for the inferred value between different datasets, indicating the parameters are less constrained in these cases (results not shown). Additional file 1, *Figure S1 *shows a typical gradient descent run using the one of the datasets generated with (*k*_1_, *k*_2_) = (0.03,0.6), which consists of 21 data points with a total time period of T = 40. We observed that the parameters converge very quickly during the gradient descent, typically within 20 steps for our tested random start values.

### Example 2: Prokaryotic auto-regulatory gene network

The second model we tested is a prokaryotic auto-regulatory gene network in which dimmers of a protein repress its own gene transcription by binding to a regulatory region upstream of the gene. The system, involving both transcription and translation, can serve as a simple, yet illustrative, example of gene regulation [31,39]. The reactions in the network are given below:

Here DNA, P, P_2 _and mRNA represent promoter sequences, proteins, protein dimmers and messenger RNA respectively. In this model, mRNAs and proteins are synthesized by transcription and translation processes (*R*_3 _and *R*_7_), and destroyed by degradation (*R*_4 _and *R*_8_). The proteins can form a dimmer P_2 _(*R*_5 _and *R*_6_), which binds and unbinds to DNA (*R*_1 _and *R*_2_). When a protein dimmer binds to the promoter, it represses mRNA production. Overall, the network implements a self-regulatory mechanism to control the synthesis of the protein product, suppressing the transcription when the protein product is abundant. Note that DNA_*t *_= DNA + DNA.P_2 _is a conserved quantity in the system. The rate functions of reactions are assumed to follow mass-action law with rate parameters *k*_1 _to *k*_8_, e.g. *h*_1 _= *k*_1_·*P*_2_·DNA.

We applied our algorithm to both the fully and partially observed cases. We generated 10 datasets as observations within a time window of [0 50) with (*k*_1_,⋯,*k*_8_) = (0.1, 0.7, 0.143, 0.35, 0.3, 0.1, 0.9, 0.11, 0.2, 0.1). Datasets *D*_1 _- *D*_5 _have total copy number DNA_*t *_to be 10 with the time interval between observations (Δ*t*) from 1.0 to 0.1. The other five datasets *D*_6 _- *D*_10 _are generated with DNA_*t *_= 2. Detailed information of the datasets is shown in Table 2. For the partially observed case, we assume that only three of the species, mRNA, P and P_2_, are observed. In addition, we assume that the total copy number DNA_*t *_is known to avoid systematic bias in the sampling the system. While using the same datasets in the fully observed case, we only retain the observations corresponding to mRNA, P and P_2_. Hereinafter, we denote the datasets by *D*_1_*, *D*_2_* etc.

**Table 2:** Parameter inference result for auto-regulatory gene network model (Fully observed case)

	***k***_***1***_	***k***_***2***_	***k***_***3***_	***k***_***4***_	***k***_***5***_	***k***_***6***_	***k***_***7***_	***k***_***8***_	*Average % Err*.
Datasets	*(0.1	*0.7*	*0.35*	*0.3*	*0.1*	0.9	*0.2*	*0.1)*	
*D*_1_, Δ*t *= 1.0	0.114	0.81	0.346	0.229	0.051	0.418	0.221	0.074	24.2
*D*_2_, Δ*t *= 1.0	0.094	0.72	0.435	0.344	0.052	0.485	0.265	0.119	24.2
*D*_3_, Δ*t *= 0.5	0.113	0.82	0.408	0.321	0.075	0.75	0.226	0.095	14.7
*D*_4_, Δ*t *= 0.5	0.113	0.71	0.276	0.253	0.086	0.77	0.223	0.100	11.4
*D*_5_, Δ*t *= 0.1	0.079	0.74	0.349	0.286	0.101	0.86	0.183	0.094	6.4

									
*D*_6_, Δ*t *= 1.0	0.095	0.42	0.321	0.277	0.10	0.73	0.235	0.104	12.7
*D*_7_, Δ*t *= 1.0	0.097	0.90	0.35	0.335	0.079	0.92	0.312	0.12	17.8
*D*_8_, Δ*t *= 0.5	0.120	0.40	0.52	0.38	0.092	0.998	0.215	0.081	22.9
*D*_9_, Δ*t *= 0.5	0.116	0.96	0.41	0.41	0.101	1.01	0.144	0.094	19.3
*D*_10_, Δ*t *= 0.1	0.052	0.91	0.277	0.35	0.128	0.93	0.137	0.075	25.4

* True values of parameters

Total observation time window = 50. For datasets *D*_1_-*D*_5_: DNA*t *= 10; *D*_6_-*D*_10_:DNA*t *= 2.

*Average % Err*. ≡ ⟨|*k*_*i *_- *k*_*i, true*_|/*k*_*i, true*_⟩_*i*_

For the fully observed case, the net effect reaction matrix is, shown with the corresponding reactions and species

The corresponding null set contains four elementary modes, consisting of the following four pairs of reactions: *R*_*1 *_- *R*_*2*_, *R*_*3 *_- *R*_*4*_, *R*_*5 *_- *R*_6_, and *R*_7 _- *R*_8_.

**Figure 2:**
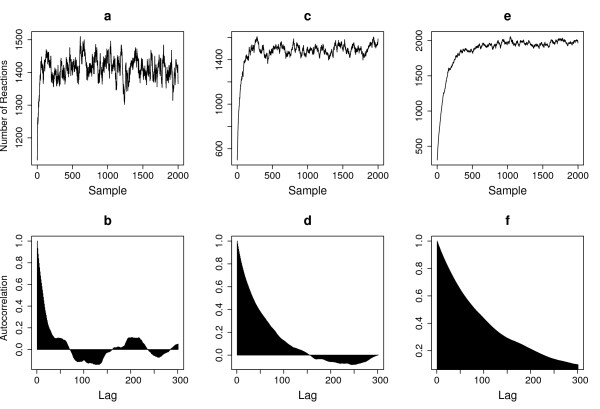
**Property of the RJMCMC sampler for the fully observed case of the auto-regulatory gene network**. Panel **a, c, **and **e **plot the total number of reactions in each of the samples generated by RJMCMC, corresponding to three different observation datasets with Δ*t *= 0.1, 0.5 and 1 respectively. Panel **b, d, **and **f **shows the autocorrelation on the number of reactions corresponding to panel **a, c, **and **e **respectively. The rate parameters used are (*k*_1_,...*k*_8_) = (0.1, 0.7, 0.143, 0.35, 0.3, 0.1, 0.9, 0.11, 0.2, 0.1).

We focus our analysis on datasets *D*_1 _- *D*_5_, of which the observation intervals range from 1.0 to 0.1. The results from datasets *D*_6 _- *D*_10 _are similar. The convergence property of the RJMCMC sampler is shown in Figure 2. It shows the RJMCMC sampler is efficient and induces good mixing for all the datasets, with convergence occurring typically after 200 samples. The correlation lengths between the samples are smaller for the more densely observed dataset (*D*_5 _with Δ*t *= 0.1). The correlation length increases for the datasets with increasing Δ*t*, suggesting the need of using larger sample size for sparse observed datasets.

We applied the stochastic gradient descent method to estimate the rate parameters given the observations. The initial parameter values were randomly chosen between 0.1 and 10. We used 5000 samples to calculate the gradient with a burn-in size of 200. The estimated parameters for each dataset are summarized in Table 2. We observed a good agreement between the estimated and true values for most of the parameters. Also we observed that there is differences in the estimation accuracy for different parameters, with some (e.g. *k*_2_, *k*_3 _and *k*_4_) showing consistently better results than others (Additional file 1, *Figure *S2). The estimation of *k*_5 _and *k*_6 _showed large deviation for the first two datasets with large observation interval (*D*_1 _and *D*_2_, Table 3), but improved with finer-sampled data. This is likely due to the faster dynamics of the two reactions (*R*_5 _and *R*_6_) than other reactions in the system.

**Table 3:** Parameter inference result for auto-regulatory gene network model (Partially observed case)

Datasets	***k***_***1***_	***k***_***2***_	***k***_***3***_	***k***_***4***_	***k***_***5***_	***k***_***6***_	***k***_***7***_	***k***_***8***_	***Average % Err***.
	*(0.1	*0.7*	*0.35*	*0.3*	*0.1*	*0.9*	*0.2*	*0.1)*	
	0.102	0.47	0.44	0.214	0.040	0.326	0.400	0.156	46.1
	0.090	0.70	0.440	0.348	0.052	0.483	0.263	0.119	24.6
	0.125	0.91	0.402	0.316	0.077	0.78	0.230	0.097	16.2
	0.188	0.64	0.413	0.250	0.072	0.64	0.43	0.196	49.9
	0.078	0.76	0.350	0.300	0.103	0.88	0.188	0.097	5.6

									
	0.108	0.41	0.303	0.247	0.131	0.955	0.214	0.107	16.5
	0.079	0.56	0.383	0.332	0.073	0.82	0.228	0.099	14.0
	0.123	0.41	0.55	0.386	0.079	0.87	0.213	0.085	24.5
	0.103	0.81	0.419	0.421	0.102	1.04	0.142	0.097	16.0
	0.075	0.75	0.37	0.30	0.13	0.96	0.25	0.11	13.7

* True values

Datasets *D*_1_*-*D*_10_* correspond to *D*_1_-*D*_10 _in Table 3 but with speices mRNA, P and P_2 _only.

*Average % Err*. ≡ ⟨|*k*_*i *_- *k*_*i, true*_|/*k*_*i, true*_⟩_*i*_

**Figure 3:**
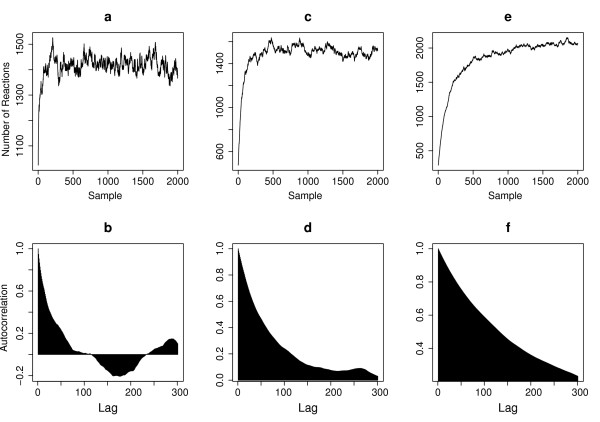
**Property of the RJMCMC sampler for the partially observed case of the auto-regulatory gene network**. Panel **a, c, **and **e **plot the total number of reactions in each of the samples generated by RJMCMC, corresponding to three different observation datasets with Δ*t *= 0.1, 0.5 and 1 respectively. Panel **b, d, **and **f **shows the autocorrelation on the number of reactions corresponding to panel **a, c, **and **e **respectively. The rate parameters used are (*k*_1_,...*k*_8_) = (0.1, 0.7, 0.143, 0.35, 0.3, 0.1, 0.9, 0.11, 0.2, 0.1). Only three species, mRNA, P and P_2_, are observed.

Next we applied our algorithm to the partially observed case. The convergence property of the RJMCMC sampler for the partially observed case is shown in Figure 3. Compared with the fully observed case, the autocorrelation length in the partially observed case is typically longer, but the RJMCMC sampler can still induce good mixing for each dataset with adequately large sample size.

The parameter inference results are summarized in Table 3. We found that the accuracy of the inferred parameter varies for different datasets. For the densely observed dataset *D*_5_*, the estimated values of all eight parameters are similar to those in the fully observed case and close to the true values. But for more sparsely observed datasets, the average percent of error of the inferred parameters increases significantly (compared to the fully observed case) for some of the datasets (*D*_1_* and *D*_4_*). The parameters *k*_1 _and *k*_2_, which are associated with the unobserved species, showed large variations between different datasets. In general, the results showed that parameter inference with partially observed data is more difficult than the one with fully observed data, and to achieve good estimation accuracy, more observations with small observation intervals will be needed.

Additional file 1, *Figure S3 *shows the changes of parameters and gradients during one gradient descent run for the most sparsely observed dataset *D*_1 _and *D*_1_* with the dataset (copy number of each species) shown in Additional file 1, *Figure S4*. Some of the parameters (e.g. *k*_2 _and *k*_6_) showed slow convergence during gradient descent in both fully and partially observed cases, which may reflect a flat likelihood surface in the corresponding parameter direction and an inherent difficulty in identifying these parameters.

## Discussions

Recently there has been a growing interest in describing biological systems using stochastic models. However, most of the parameters in the stochastic models are unknown and difficult to measure. In this paper we described a maximum likelihood method to infer the parameters of a stochastic kinetic model directly from observations. Our method works by estimating the gradient of the likelihood function first, and then searching for an optimal solution by iteratively updating the parameters along the gradient descent direction. We developed a general RJMCMC algorithm to sample the latent reaction path in a constrained setting, where the reaction path has to match the observations given at the two ends of a time interval. The sampled reaction paths are used to calculate the gradient of the likelihood function using a formula that we derived. The availability of the gradient information makes it possible to develop other algorithms to solve the maximum likelihood estimation problem, in addition to the steepest descent method that we implemented. Furthermore, the availability of the gradient information also enables other possible applications such as parameter sensitivity analysis, which has already attracted considerable interest in deterministic modeling [50,51].

Our method is significantly faster than the SML method [34], which is also a maximum likelihood based parameter inference method. SML uses two steps to estimate parameters. First, it estimates the transition density on reaction species numbers after a given time interval, using a SSA-based sampling methods. The estimated transition density is then used to calculate the likelihood function. Because the gradient of the likelihood function is not directly available, SML uses a genetic algorithm to solve the maximum likelihood problem. Comparing the SML and our method for the birth-death example, we tested the CPU time used to generate a new sample for both methods, eg. SSA for SML (unconstrained) and RJMCMC for SGD, which is approximately the same. However, SML uses 3 × 10^4 ^evaluations of transition density to reach a solution. By contrast, SGD typically requires less than 20 evaluations of the gradient before convergence. If we ignore the computational time of the gradient descent steps, overall SGD achieves a reduction of computational time by an order of 10 ^3 ^compared to SML.

In terms of accuracy, our approach, based on exact sampling, should be less biased than approximation-based methods. In this regard, we compared SGD to the method by Golightly *et al. *[31], who used a diffusion approximation to calculate the transition density. Comparing the results obtained by both methods on the same datasets (in courtesy of Dr. Golightly), we note that the estimated values for *k*_1 _and *k*_2 _by our method are closer to the true results in all three test datasets while the result from [31] are biased toward low values, although the estimates for other parameters from the two methods are similar (Additional file 1, Table S1). Interestingly, *k*_1 _and *k*_2 _are associated with low copy number of reaction species (DNA and DNA.P_2_). We also tested the method in [31] with the datasets of DNA_*t *_= 2 and found that the algorithm gives worse results, especially for the first two parameters (result not shown). This reflects the advantage of our method and possibly the limitation of the diffusion approximation, which assumes that the values of the hazard functions are approximately constants between two observation/latent states. This assumption is not valid if the copy numbers of species are small in the reactions. For example, in case of DNA_*t *_= 1, reactions *R*_1 _and *R*_2 _can only happen alternatively and this clearly violates the approximation assumption.

Our method is closely related to the full Bayesian approach proposed by Boys *et al. *[33] as both methods use RJMCMC to sample the reaction process. Comparing to the method by Boys *et al*., our method offers two improvements. First, we provide a general method for RCMCMC sampling, which can be applied to an arbitrary biochemical reaction system, while the previous method is only tailored to a specific reaction system (more specifically, the Lotka-Volterra system). Second, the gradient-based method is significantly faster than the full Bayesian method as sampling the parameter space is often computationally challenging. However, the Bayesian approach offers certain advantage over the maximum likelihood method in that it provides a posterior distribution of the parameters rather than just an optimal solution. In this regard, we note that the general RJMCMC sampling method we developed can be easily extended for Bayesian inference after introducing additional Metropolis-Hasting steps for sampling parameters.

## Conclusion

In this paper, we proposed a new algorithm for inferring rate parameters in stochastic models and tested it using simulated data. Although few biological systems with measurements of species numbers across multiple time points are currently available, this type of data will likely become more common in the future, given rapid advances in single cell measurement technology [9,52,53]. The method could also be applied to cell colony data, e.g. in [54], which proposed some interesting models involving stem cell homeostasis process. As we observed, the current RJMCMC sampler can be inefficient in some cases with large observation intervals. One possible improvement of the current algorithm is to use more efficient sampling algorithm, for example, the blocking updating scheme in [33]. It is evident that significant challenges remain in dealing with true biological systems, including measurement noise, uncertainty in models, and sparsity of the data. However, studying stochastic systems with parameters inferred directly from data should be able to lead to a better understanding of the systems than the current approach of manually setting these parameters.

## Authors' contributions

The study was initially conceived by *EM *and *YW*, and later extended by *SC *and *XX*. *YW *implemented the algorithm and carried out most of the computational analysis. *YW*, *SC *and *XX *wrote the paper. All authors read and approved the final manuscript.

## Appendix

### Derivation of the formula on calculating the gradient of the likelihood function

Consider the time interval [*t*_*s*_, *t*_*s*+1_] with full observations available at the start and the end of the interval, denoted by *X*(*t*_*s*_) and *X*(*t*_*s*+1_) respectively. To calculate the likelihood function *L*(*X*(*t*_*s*_), *X*(*t*_*s*+1_);Θ), we discretize the time interval into *N *subintervals and denote the system states at these discrete points by {*X*^*i*^|*i *= 0,⋯,*N*}, where *X*^0 ^= *X*(*t*_*s*_) and *X*^*N *^= *X*(*t*_*s*+1_) are two observations, and all other *X*^*i *^s are intermediate states and not directly observable.

After the discretization, the likelihood function becomes, after using the Markov property of the process(A.1)

For sufficiently large *N*, from the master equation Eq. (1.3), the conditional probability can be approximated by , where *dt *= (*t*_*s *+ 1 _- *t*_*s*_)/*N *and *δ*_*X', X *_is the Kronecker delta function.

We are interested in the gradient of the likelihood function instead of calculating the likelihood function explicitly. So we take the partial derivative of *L*(*X*(*t*_*s*_), *X*(*t*_*s*+1_);Θ) *w.r.t*. the parameters,(A.2)

Note that when *N *→ ∞,

Therefore(A.3)

where *T*_k _is the time duration of the system at state *k*, and *N*_*k, k' *_is the number of transitions from state *k *to *k' *occurred during the interval. Both *T*_k _and *N*_*k, k' *_are random variables, and can be viewed as the sufficient statistics of the model. *E*[·] represents the expectation of the random variables. For hazard functions of the form (1.5),  and , thus equation (2.1) and (2.4) follow.

The formula can also be derived by the time ordered product expansion described in [55] without resorting to time discretization, as shown below. This result (Eq. A.3) suggests that the gradient of the likelihood function can be calculated by estimating the expectation of the two sufficient statistics. Note that the formula is quite general and holds for any stochastic system obeying the master equation (Eq. 1.3).

### Derivation of the RJMCMC algorithm based on the time ordered product expansion of master equation

Representing the probability vector of system state as , the master equation (Eq. 1.3) can be written in a compact matrix form, using column vector notation (in this section),

where *H *is the time evolution matrix, of which the elements are uniquely determined by the stoichiometry matrix and the hazard functions.

The formal solution of master equation is

The probability of system evolving from a particular start state to an end state is given by the corresponding elements of the probability matrix . The time evolution matrix *H *is usually an infinite dimension matrix, for there are usually no upper bound for the species numbers.

The time ordered product expansion (TOPE) formula, which originates from quantum field theory, is useful to make series expansion of the matrix exponential. If we decompose the evolution matrix into two parts, *H *= *H*_0 _+ *H*_1_, the TOPE formula gives [55]

where *τ*_0 _= *t*_1_, *τ*_1 _= *t*_2 _- *t*_1 _*etc*. A proper choice is to decompose *H *into diagonal and off-diagonal matrices *H *= *Ĥ *- *D*, i.e. *H*_0 _= - *D *and *H*_1 _= *Ĥ*. This leads to the TOPE formula

where *D *represents the diagonal part (non-negative) and *Ĥ *is the off-diagonal part of the matrix. The terms inside the integral, conditioned on a given Markov jump process, is the likelihood (or probability density) of the process. In case of reaction systems, a process corresponds to a set of reaction events. Thus the *k*th order integration gives the total probability of all reaction events with *k *reactions. We note that the TOPE formula provides a possible way to estimate the matrix exponential (probability matrix) by Monte Carlo integration by randomly casting reaction events and summing up the likelihood.

In the fully observed case, the likelihood function is the product of the likelihood of each sub-interval,

The likelihood for each sub-interval can be denoted as

where *τ *= *t*_*s*+1 _- *t*_*s*_, and in the last step of the above equation we approximate the integration by a Monte-Carlo integral with *π*(Ξ({*r*_*i*_, *t*_*i*_})|*θ*_*r*_) to be the likelihood of latent process Ξ({*r*_*i*_, *t*_*i*_}) (see Eq. 2.3) which is constrained by start/end observation (*x*_*a*_(*t*_*s*+1_) and *x*_*a*_(*t*_*s*_)) and  in which *n*_*r *_is the number of reaction type *r*. *V*_*k *_is the multiplication of two parts: the first part arises from the simplex integration of the time variables, which can be viewed as the measure of integration space when we convert the integration to summation; the second part is a combinatorial factor resulting from the permutation invariance of the same reaction type in a given reaction path.

Recalling that in the RJMCMC algorithm, we generate samples with different number and type of reactions via the Metropolis-Hasting steps. The ratio between *π*(·)/*V*_*k *_of two samples gives the same acceptance probability as in Eq. (2.5).

Assuming all the reaction follows mass-action law, we can derive the gradient of the likelihood function using TOPE formula. We can write *Ĥ *and *D *matrix in terms of the component of each reaction type, i.e. . Thus

We define , which is the branching ratio for reaction *r *in state *X'*. Then

Thus,

which gives the gradient formula in Eq. (2.1), (2.4) and (A.3), since the average of a frequency gives the probability.

## Algorithm 1. Pseudo-code of RJMCMC algorithm for fully observed case

*Input observations *{*n*(*t*_*s*_)} *and generate initial path for each interval using GLPK;*

*Calculate the null set *{*q*'_*k*_} *with the net-effect reaction matrix A;*

***for ****iter = 1: maxiteration*

   *Randomly choose an elementary mode q*_*k*·_;

   ***for ****i = 1: number of time intervals*

      *Randomly choose a move type p and update the reaction path in sub-interval [t_i_, t_i+1_) according to *;

      *Calculate the number of each reaction type r*_*m*_

      *if min(r*_*m*_*) == 0, AP = 0; break*

      *else*

         ***for ****j = 1: J (total number of reactions within the interval)*

            *if **is negative for any a *∈ (1,2,...*K*),

               *AP = 0; break*

            *else*

            *Calculate the intermediate species number after the reaction: *

            *endif*

         ***endfor***

         *AP = min(1, AR*_*ip*_*);*

      *endif*

      *if AP > rand(0,1)*

         *Accept the new path;*

      *endif*

   ***endfor***

*endfor*

## Algorithm 2. Pseudo-code of RJMCMC algorithm for partially observed case

   *Input observations *{*n*_Γ_(*t*_*s*_)} *and randomly specify state for the unobserved species, generate initial path for each interval with GLPK; *

   *Calculate the null set *{} *using the partial reaction matrix A*_*p*_*;*

   ***for ****iter = 1: maxiteration*

      ***for ****i = 1: number of time intervals*

         *Randomly choose an elementary mode **and a move type p; Update the reaction path in sub-interval (t*_*i *_*, t*_*i*+1_*) according to **;*

         *Calculate the number of each reaction type: **, AP = 0; break*

         *else*

            ***for ****j = 1: number of reactions within the ith interval*

               *if **is negative for any species a AP = 0; break*

               *else*

               *Calculate the intermediate species number after the reaction: **;*

               *endif*

            ***endfor***

         *endif*

         *if x*_*a, J *+ 1 _== *x*_*a*_(*t*_*t*+1_)

            

         *else*

            *Update the second interval via complementary move p';*

            *Calculate the number of each reaction type: *

               *if **, AP = 0; break*

               *else*

                     ***for ****j' = 1: number of reactions within the (i + 1)th interval*

                     *if **is negative for any species a AP = 0; break*

                     *else*

                     *Calculate the intermediate species number after the reaction: **;*

                     *endif*

                  ***endfor***

               *endif*

            *Calculate  for the new path and the acceptance probability *;

         *endif*

      *if AP > rand(0,1)*

            *Accept the new path;*

      *endif*

   ***endfor***

*endfor*

## Algorithm 3: Stochastic gradient descent algorithm

   *Input: time-course data *

   *Output: set of inferred parameters *{*θ*_*r*_}

   *1. Initialize the reaction path using GLPK and set initial values of rate parameters;*

   *2. Sample the latent paths for the entire observation interval with reversible jump MCMC*

      -*For fully observed case: sample latent paths for each interval s ∈ (0, m-1) using Algorithm 1;*

      -*For partially observed case: sample latent paths for each paired intervals and separately for the first interval using Algorithm 2;*

      *Calculate the gradient of each sample path **according to Eq. (2.3) after burn in;*

   *3. Calculate the gradient by averaging over sample paths;*

   *4. Update parameter values by gradient descent step:*

      *for all r, where η is the step size;*

   *5. If convergence condition (to be specified) is not satisfied, return to step 2*.

## Supplementary Material

Additional file 1 **Supplementary figures and tables**. This file contains Supplementary Figure S1-S4, Table S1. Click here for file
