# Supplementary material for: Parameter inference for discretely observed stochastic kinetic models using stochastic gradient descent
Source: BMC Syst Biol. 2010 Jul 21;4:99. doi: 10.1186/1752-0509-4-99 (PMC2914651; doi:10.1186/1752-0509-4-99)
Supplement: Additional file 1 — Supplementary figures and tables. This file contains Supplementary Figure S1-S4, Table S1. [file 1752-0509-4-99-S1.DOCX]

# Supplementary Material

# Parameter inference for discretely observed stochastic kinetic models using stochastic gradient descent

Yuanfeng Wang^1,4^, Scott Christley^2,4^, Eric Mjolsness^3,4^ and Xiaohui Xie^3,4†^

^1^Deparment of Physics and Astronomy, ^2^Department of Mathematics, ^3^Department of Computer Science, ^4^Center for Complex Biological Systems, University of California, Irvine, CA 92617, USA

^†^ Corresponding Author

Email addresses:

YW: [yuanfenw@uci.edu](mailto:yuanfenw@uci.edu)

SC: [scott.christley@uci.edu](mailto:scott.christley@uci.edu)

EM: [emj@uci.edu](mailto:emj@uci.edu)

XX: [xhx@uci.edu](mailto:xhx@uci.edu)

**
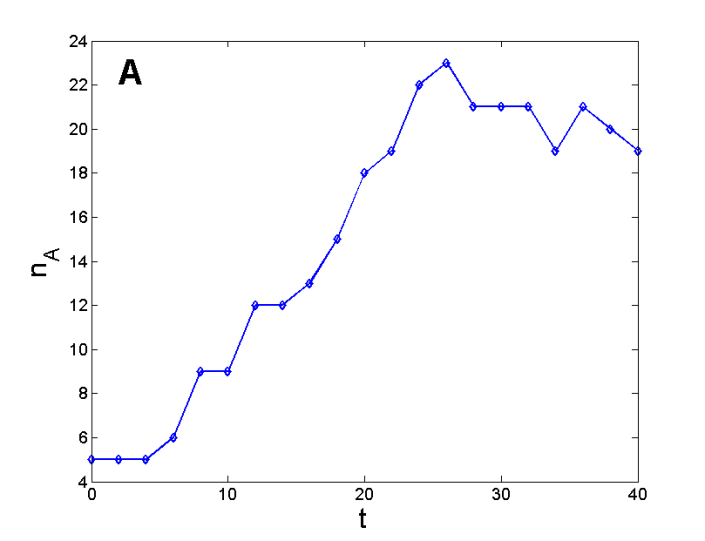

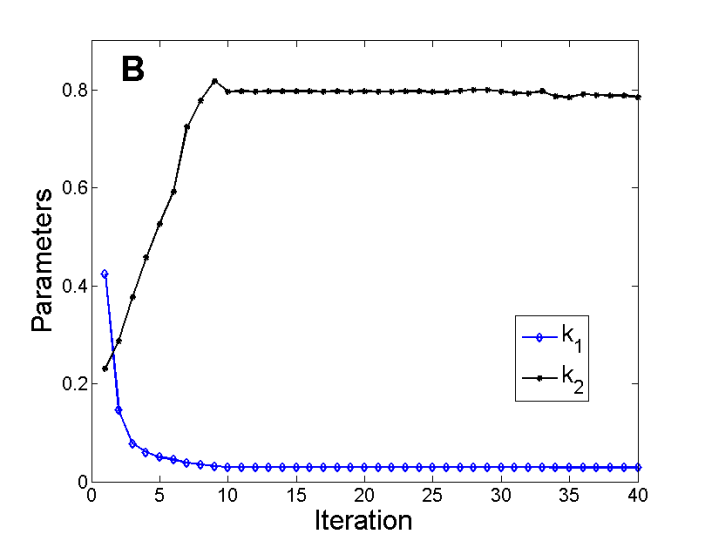

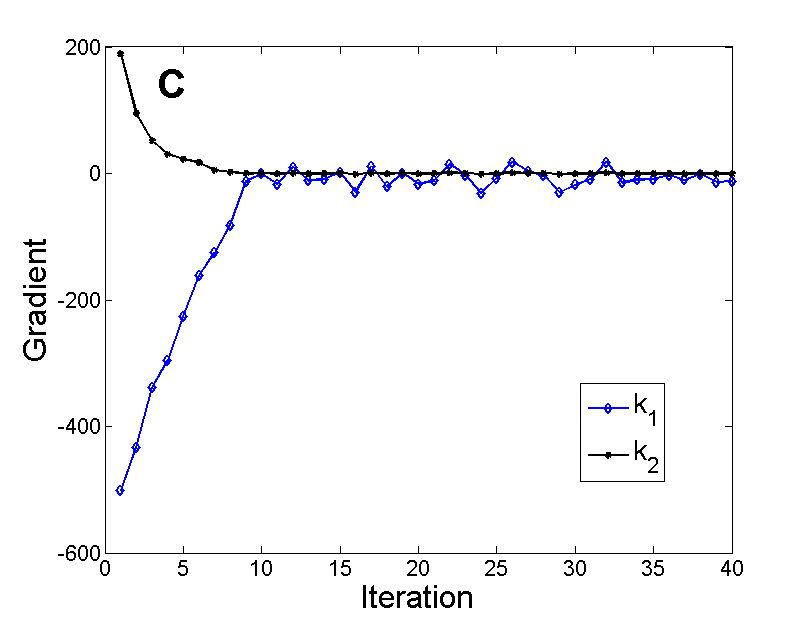
**

**Figure S1 - Gradient descent run for the birth-death process. (a)** shows an observation dataset (the first dataset in Table 2) generated by SSA with initial population = 5 and parameter value (*k*_1_, *k*_2_) = (0.03, 0.6). **(b)** The value of parameters (*k*_1_, *k*_2_) during one gradient decent run for the data set in (a). **(c)** The value of gradients corresponding to each of the parameters.

**
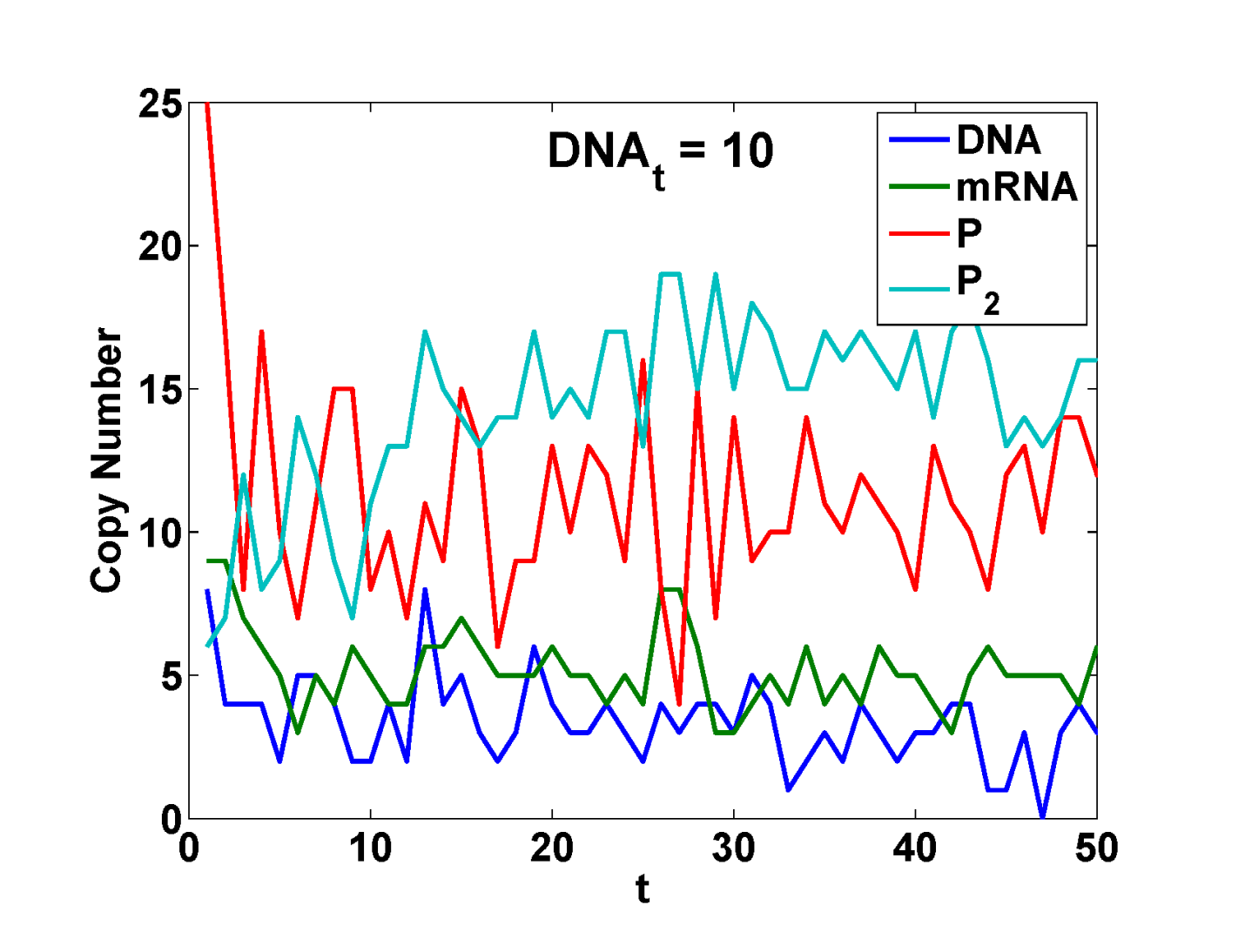
**

**Figure S2 – Plot of the copy number of each species in dataset *D*_1_ (**$\boldsymbol{\Delta t=1}$**) with species indicated by different colors (in the legend).**

**
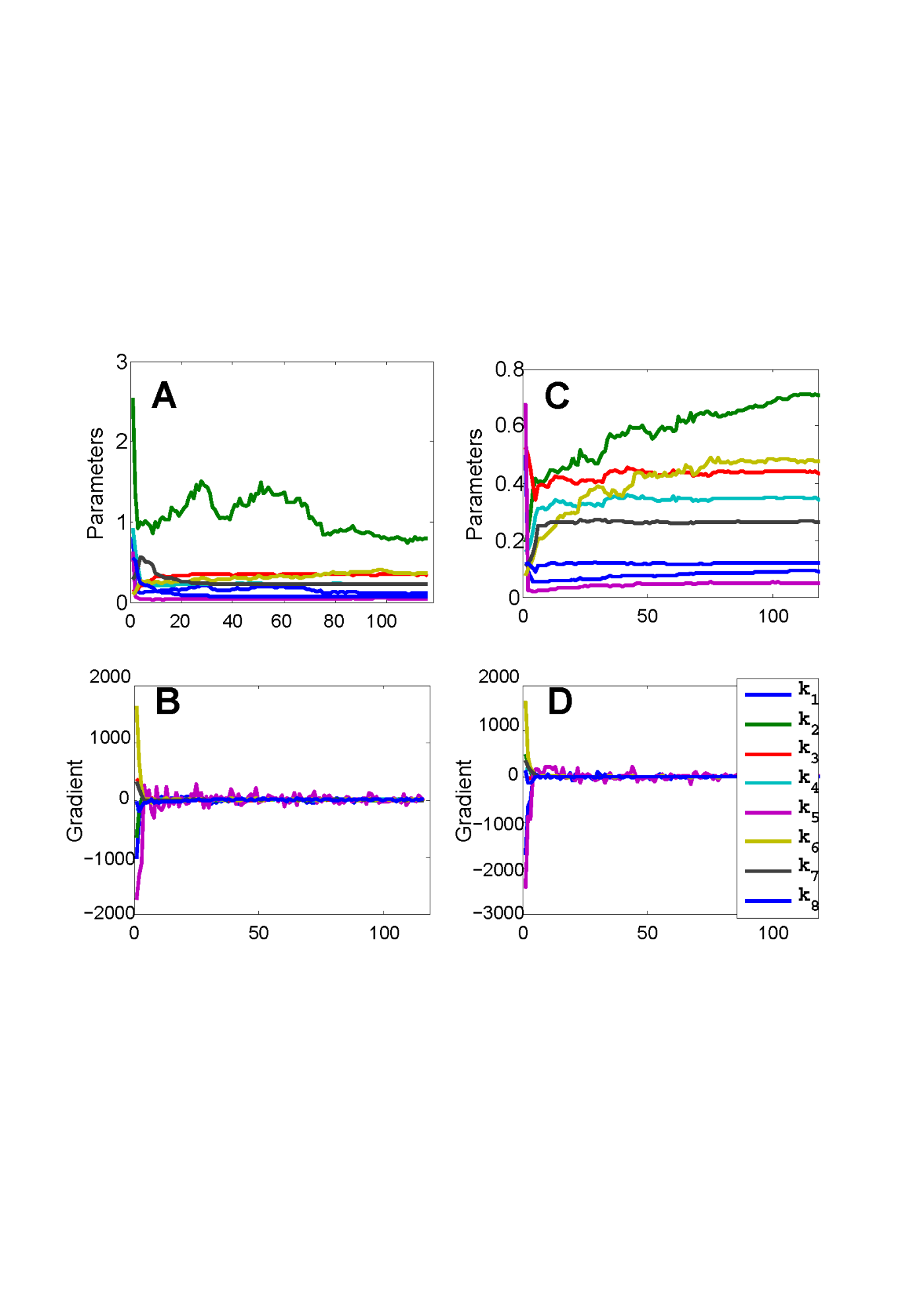
**

**Figure S3 - Gradient descent run for the auto-regulatory gene network model.** Panel **a** and **c** plot the values of eight parameters (*k*_1_ to *k*_8_) in one gradient decent run using the dataset *D*_1_ (the fully observed dataset) and *D*_1_^*^ (the partially observed dataset) respectively. Panel **b** and **d** show the corresponding gradients of parameters for **a** and **c** respectively. Parameters and corresponding gradients are indicated with different colors.


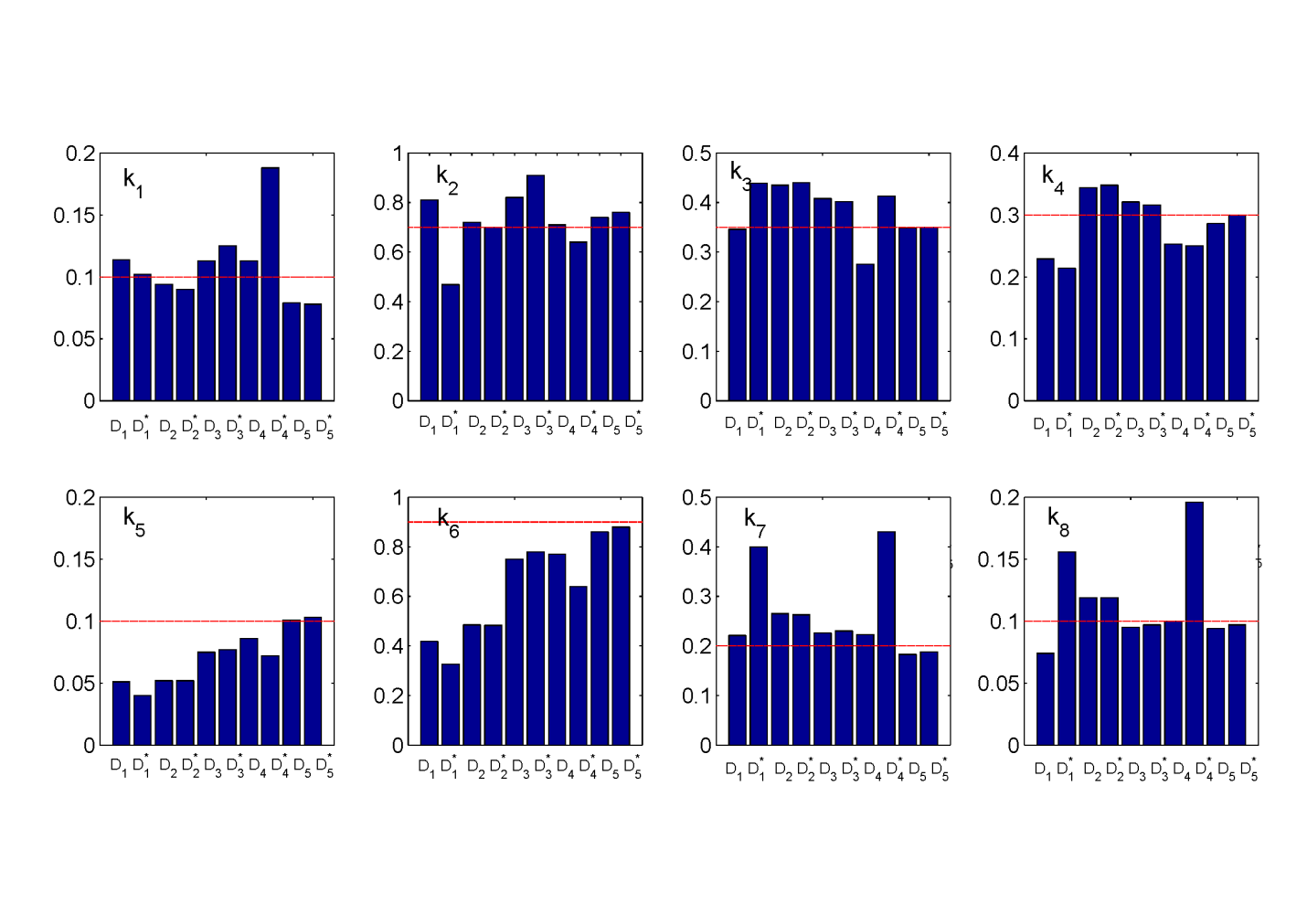


**Figure S4 – Estimated rate parameters in the auto-regulatory gene network.** Bar plots of the estimated values of rate parameters from different observation datasets. Each panel corresponds to parameters *k*_1_ to *k*_8_ respectively. Horizontal lines indicate the true values of parameters.

**Table S1.** **Comparison of the inferred parameter values of two methods with the datasets (D_1_-D_3_) in Golightly *et al.* 05 [31]**

|  | *k_1_* | *k_2_* | *k_3_* | *k_4_* | *k_5_* | *k_6_* | *k_7_* | *k_8_* |
| --- | --- | --- | --- | --- | --- | --- | --- | --- |
| *Dataset1* | ^1^0.086 | 0.673 | 0.35 | 0.287 | 0.061 | 0.59 | 0.242 | 0.14 |
|  | ^2^0.064 | 0.47 | 0.36 | 0.288 | 0.043 | 0.475 | 0.25 | 0.143 |
| *Dataset2* | ^1^0.093 | 0.63 | 0.401 | 0.305 | 0.069 | 0.60 | 0.224 | 0.115 |
|  | ^2^0.058 | 0.36 | 0.285 | 0.24 | 0.048 | 0.477 | 0.285 | 0.121 |
| *Dataset3* | ^1^0.053 | 0.519 | 0.346 | 0.295 | 0.028 | 0.511 | 0.162 | 0.088 |
|  | ^2^0.052 | 0.346 | 0.416 | 0.321 | 0.044 | 0.488 | 0.213 | 0.115 |
| ^3^Percent error | 22.7 | 13.2 | 5.2 | 2.6 | 47.0 | 37.0 | 17.3 | 22.8 |
|  | 42.0 | 44.2 | 13.4 | 10.3 | 55.0 | 46.7 | 24.7 | 26.4 |
| 1 Inferred parameter values by our method  2 Mean values of the inferred parameters from Table 1 of [31]  ^3^ percent error is the averaged value over three datasets | | | | | | | | |
